# Militarism and globalization: Is there an empirical link?

**DOI:** 10.1007/s11135-017-0525-4

**Published:** 2017-06-16

**Authors:** Manuchehr Irandoust

**Affiliations:** Department of Economics and Finance, School of Business Studies, Kristianstad University, 291 39 Kristianstad, Sweden

**Keywords:** Militarism, Globalization, Causality, Military spending, F14, F51, F62

## Abstract

Despite the fact that previous studies have extensively investigated the causal nexus between military expenditure and economic growth in both developed and developing countries, those studies have not considered the role of globalization. The aim of this study is to examine the relationship between militarism and globalization for the top 15 military expenditure spenders over the period 1990–2012. The bootstrap panel Granger causality approach is utilized to detect the direction of causality. The results show that military expenditure and overall globalization are causally related in most of the countries under review. This implies that countries experiencing greater globalization have relatively large increases in militarization over the past 20 years. The policy implication of the findings is that greater military spending by a country increases the likelihood of military conflict in the future, the anticipation of which discourages globalization.

## Introduction

The literature on military spending and its effect on economic variables can be classified into two hypotheses. The first hypothesis deals with military spending-growth nexus (e.g., Hooker and Knetter 1997; Alptekin and Levine 2012: Dunne and Tian 2013; Yilgör et al. 2014; Dimitraki and Ali 2015). The second hypothesis considers military spending-income inequality nexus (e.g., Ali 2012; Kentor et al. 2012; Elveren 2012; Wolde-Rufael 2016).

Previous studies have extensively focused on the above-mentioned areas and they have not considered globalization-military spending nexus. There are two opposite effects with regard to globalization. On the one hand, with the opening of borders to trade and foreign investment, globalization creates opportunities and pressures for domestic firms to innovate and improve their competitive position. Many of these pressures and opportunities operate through increased competition and linkages with foreign firms. Globalization’s agenda is to improve institutions; to promote competition and efficiency; to implement policies that raise productivity; and to create economic, political, and social stability. On the other hand, globalization, due to market failure argument, might result in instability, making countries more vulnerable to external shocks, reducing growth and increasing government spending when dealing with the undesired effects of globalization.

Inevitably, national security requires dealing with a wide range of issues. These include those discussed in the traditional security sphere as well as understanding the changing international economic and political environment. Especially, when the current international environment is surrounded by increasingly complex issues related to international economics, national security and foreign policy, it is difficult to come with any proposition without considering interaction between these issues. In other words, there seems to be a growing tendency for governments to view issues of international economics through the prisms of national security and foreign policy as well as though the default one of economic policy (Acemoglu and Yared 2010).

Globalization makes production factors, goods, and services increasingly mobile across national boundaries. Openness to international trade, finance, foreign direct investment (FDI) and technology are choices that countries make. It has been argued that the end of the previous 19th century wave of globalization was disappointment which accelerated by the Great Depression and the rise of nationalism, militarism and international conflict (e.g., Findlay and O’Rourke 2007; Glick and Taylor 2010; Acemoglu and Yared 2010). Globalization, which is determined by political and economic decisions of nation states, has political limits, and that these limits are subject to nationalism and militarism (Acemoglu and Yared 2010). Despite the increasing role of globalization, evidence reveals that nationalism and militarism are strong around the world, in countries ranging from the United States to China, Russia and India (e.g., Kagan 2008).

However, the aim of this paper is to examine the relationship between militarism and globalization for the top 15 military expenditure spenders over the period 1990–2012. Militarism, which is measured by military spending, is a useful proxy since it might itself impact economy in general. This is because it contributes to tensions or leads to conflict between countries, thus other economic variables (such as employment, FDI, and trade) are affected. There is the steady rise of globalization and military expenditures (or the size of military personnel) across a large number of countries over the past two decades. Military spending, after declining for a number of years, started increasing from the mid-1990s onwards. This pattern indeed reveals that there might be evidence pointing to a strengthening in nationalist sentiments and militarism.

The main contribution of this paper is to show how military spending, as a proxy for nationalist sentiment and militarism, is associated with globalization. The evidence shows that there might be political and military limits to, and dangers against, globalization as argued by Acemoglu and Yared (2010). To the best of the author’s knowledge, this is the first study to examine the relationship between globalization and militarism by using the bootstrap panel Granger causality approach.

This paper is reexamining a growing literature in international relations and International Economics on the so-called liberal theory which argues that greater trade makes war less likely. Empirical studies estimate the effect of trade on war and of war disruptions on trade (Gasiorowski and Polachek 1982; Holsti 1991; Barbieri 1996; Levy and Ali 1998; Russett et al. 1998; Barbieri and Schneider 1999; Polachek et al. 1999; Oneal and Russet 1999; Russett and Oneal 2001; O’Rourke and Sinnott 2001; Gartzke and Li 2003; Martin et al. 2008; Hegre et al. 2010; Acemoglu and Yared 2010).

The empirical evidence, however, provides mixed and conflicting results. While some studies have found, heightened trade has inhibited military conflict (e.g., Gasiorowski and Polachek 1982; Oneal et al. 1996; Russett et al. 1998; Oneal and Russet 1999; Russett and Oneal 2001), other studies have pointed out how the expansion of major power trade networks within a discriminatory, mercantilist framework aggravated commercial rivalries and sometimes stimulated armed conflict (e.g., Holsti 1991; Barbieri 1996; Barbieri and Schneider 1999; Levy and Ali 1998). Thus, trade has expanded within two different policy contexts: initially embedded in a more state-directed and imperialist environment during the mercantilist era and later within a more liberal economic regime. Since previous studies about the relationship between attitudes towards trade and nationalist sentiment have not generated any consensus, it is worth to provide some new insights on these issues.

The remainder of this paper is organized as follows: Sect. 2 outlines theoretical considerations, Sect. 3 reviews some stylized facts about military spending and globalization, Sect. 4 introduces data and methodology, Sect. 5 presents empirical evidence, and finally, Sect. 6 offers conclusion.

## Theoretical considerations

Much of the literature on interdependence and military conflict claims that open international markets and intense economic exchange reduce interstate hostilities.^1^ Liberals have been the strong supporters of this thesis and have mentioned a variety of different causal mechanisms in developing it (Mansfield and Pollins 2006). One argument is that economic exchange and military conquest are substitute means of acquiring the resources needed to promote political security and economic growth (e.g., Staley 1939). As trade and FDI increase, there are fewer incentives to meet these needs through territorial expansion, imperialism, and foreign conquest (Rosecrance 1986).

Liberals also emphasize that economic integration increases contact and promotes communication between private and public actors in different countries. This, in turn, is expected to promote cooperative political relations (Viner 1951; Doyle 1997). Many liberals also suggest that economic openness creates efficiency gains that, in turn, cause private traders and consumers to be dependent on foreign markets. These actors put pressures on public officials to avoid military conflicts because political antagonism may break apart economic relations among participants and jeopardize the gains from trade. Public officials accept such demands since they rely on societal actors for political support and have an interest in supporting their country’s economic performance.

The “liberal peace” view in political science—traced back to Montesquieu, Kant, and Angell—emphasizes that mutual economic interdependence can be a conduit of peace. It suggests that a higher degree of economic interdependence limits the incentive to use military force in interstate relations. For example, a state more trade-dependent is less likely to have a conflict with a partner because of the larger opportunity cost associated with the loss of trade. Business elites (who gain most from an increased economic interdependence) will also lobby the state to restrict the use of military force against major trading partners. Thus, an important element of the liberal position is that a liberal economic order generates a substantial and positive contribution to the maintenance of international security. Liberals also claim that barriers to international economic activity stimulate conflicts of interest that can lead to political-military discord (Viner 1951).

However, the liberal view has been criticized by mercantilists and many realists who suggest that unrestricted economic exchange can weaken national security. The gains from trade often do not accrue to national states proportionately and that the distribution of these gains can affect interstate power relations (Hirschman 1980). Shifting power relations, in turn, are widely interpreted as a potent source of military conflict (Gilpin 1981; Levy 1989; Mearsheimer 1990).

However, the extent to which trade partners are subject to their economic relationship often varies substantially among the constituent states. If one partner is dependent on a trading relationship much more heavily than another partner, the costs associated with attenuating or severing the relationship are far lower for the latter than the former state. It has been argued that asymmetric economic interdependence could lead to negative consequences in a country (such as exploited concession and threatened national autonomy), thereby creating interstate tensions and conflicts (Dos Santos 1970; Keohane and Nye 1973). Under these circumstances, trade may have negligible effect to avoid the less dependent state from initiating hostilities.

Another challenge to the liberal argument stems from the fact that states have political reasons to minimize their dependence on foreign trade and that military expansion offers some options to achieve this goal. Thus, as trade flows and the extent of interdependence increase, so do the incentives for states to take military actions to reduce their economic vulnerability (Gilpin 1981; Liberman 1996; Mansfield and Pollins 2006). Furthermore, an increase in trade leads to an increase in economic issues over which disputes can emerge. This is because close interdependence implies closeness of contact and raises the prospect of at least occasional conflict (Waltz 1970). As such, heightened interdependence may actually lead to aggressively hostile or warlike attitude.

Many studies claim that hostilities come largely from variations in the distribution of political-military capabilities and that power relations underlie any apparent effect of economic exchange on military antagonism. Such economic interdependence among the major powers was significant prior to World War I but far less extensive prior to World War II. This is often regarded as evidence that such ties have no significant impact on armed conflict when core national interests are at stake (Mansfield and Pollins 2006).

Finally, Martin et al. (2008) argue that countries more open to global trade have a higher probability of dyadic conflict because multilateral trade openness reduces bilateral dependence to any given country and, thus, lowers the opportunity cost of military conflict. Their model assumes that a bilateral military conflict between countries destroys a substantial part of the “effective labor” in them, while it increases both bilateral and multilateral trade costs. On the one hand, higher multilateral trade offsets welfare loss from decrease in effective labor during a bilateral conflict, thus reducing the opportunity cost of bilateral conflict. On the other hand, higher multilateral trade increases the opportunity cost of the bilateral conflict by raising multilateral trade costs. They further assume that the increase in multilateral trade costs following a conflict is small enough compared with the welfare loss due to the decrease in the number of consumption varieties that stem from the loss in effective labor.

Generally speaking, the dispute on the interrelationship between globalization and conflict is largely unresolved and that major aspects of this nexus have not found the scientific attention they deserve. Hence, although some of the recent contributions to this literature are impressive, it is worth to point out the following problems. First, there are still no solid theoretical foundations that provide a convincing causal mechanism for the eventual link between globalization and conflict. Although the early expected utility models supported the claim that the relationship is unconditional, the more recent game-theoretic work shows that the interrelationship, if it exists at all, depends on some crucial intervening variables like enforcement or monitoring costs.

A large body of game-theoretic work examines the process by which incomplete information, uncertainty and misperception between more-or-less rationally led states make them go into war (e.g., Fearon 1995; Van Evera 1999). Morrow (2000) suggests that crisis bargaining models should be used to study the causal nexus, a suggestion which has not yet been taken up in the empirical studies. Second, most of the studies in the dependency tradition have concentrated on the effects of economic dependency on growth and have paid relatively little attention to conflict.

Thus, despite extensive debates and research about the relationship between interdependence and war (or between economic interactions and political discord), empirical studies have produced mixed results both theoretically and empirically. Although many of them reveal that high degree of interdependence inhibits war, others suggest that rising interdependence either has no deterrent effect on war or stimulates antagonism. The reasons for these divergent and mixed conclusions might be a result of methodological differences within this literature that have remained largely unexplored.

## Some stylized facts

This sections overviews some stylized facts about the real military spending (MIS) and the overall globalization (GLOB) across countries in the sample.^2^ Figures 1a, b, 2a, b, 3a, b, 4a, b, 5a, b, 6a, b, 7a, b, 8a, b, 9a, b, 10a, b, 11a, b, 12a, b, 13a, b and 14a, b plot time trends of the variables for all countries in the sample.

**Fig. 1:**
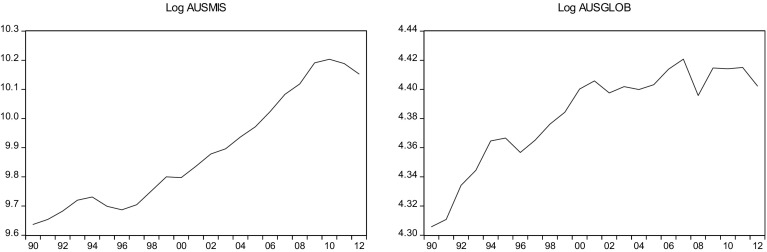
**a** Australian military spending (1990–2012). **b** Australian overall globalization index (1990–2012). *Source* KOF and SIPRI

**Fig. 2:**
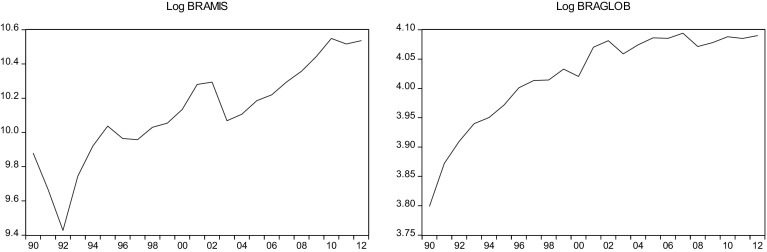
**a** Brazilian military spending (1990–2012). **b** Brazilian overall globalization index (1990–2012). *Source* KOF and SIPRI

**Fig. 3:**
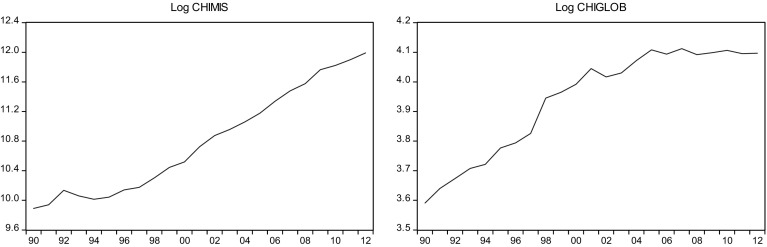
**a** Chinese military spending (1990–2012). **b** Chinese overall globalization index (1990–2012). *Source* KOF and SIPRI

**Fig. 4:**
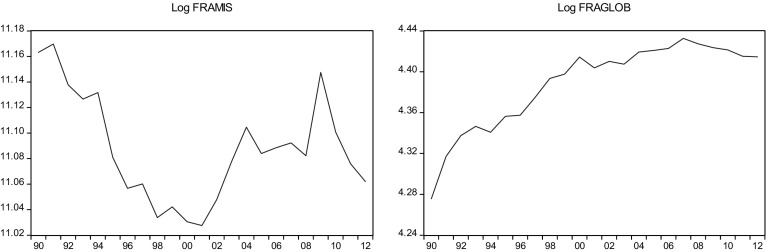
**a** French military spending (1990–2012). **b** French overall globalization index (1990–2012). *Source* KOF and SIPRI

**Fig. 5:**
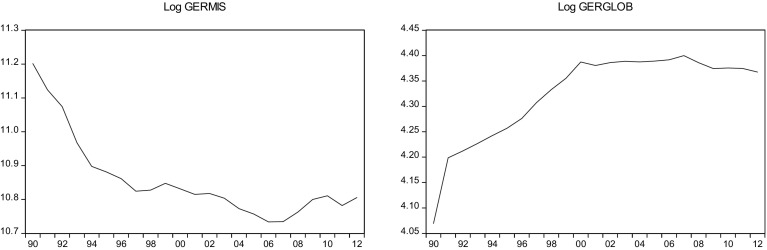
**a** German military spending (1990–2012). **b** German overall globalization index (1990–2012). *Source* KOF and SIPRI

**Fig. 6:**
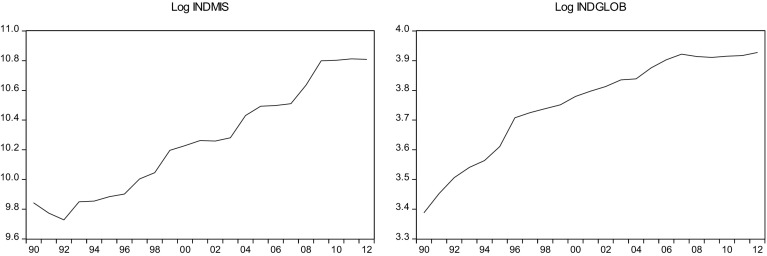
**a** Indian military spending (1990–2012). **b** Indian overall globalization index (1990–2012). *Source* KOF and SIPRI

**Fig. 7:**
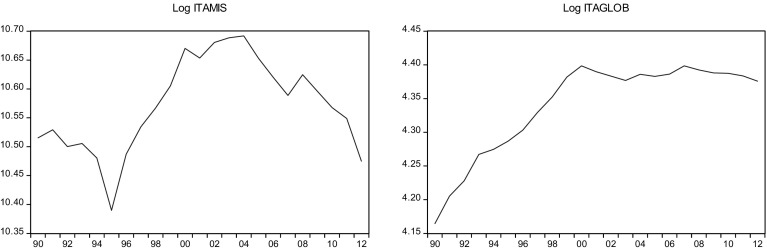
**a** Italian military spending (1990–2012). **b** Italian overall globalization index (1990–2012). *Source* KOF and SIPRI

**Fig. 8:**
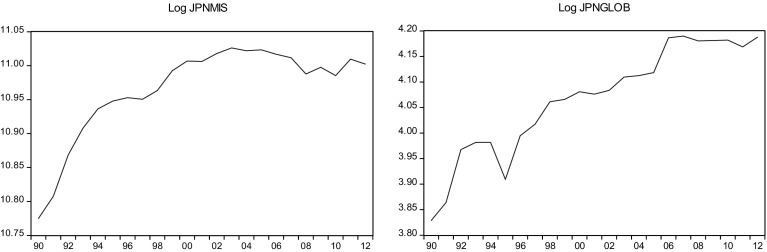
**a** Japanese military spending (1990–2012). **b** Japanese overall globalization index (1990–2012). *Source* KOF and SIPRI

**Fig. 9:**
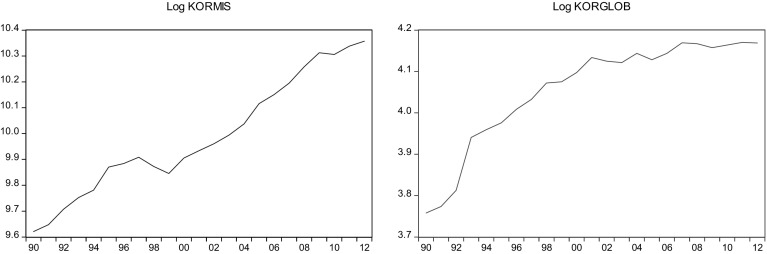
**a** Korean military spending (1990–2012). **b** Korean overall globalization index (1990–2012). *Source* KOF and SIPRI

**Fig. 10:**
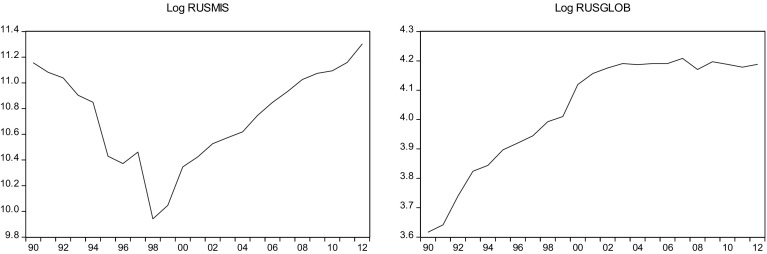
**a** Russian military spending (1990–2012). **b** Russian overall globalization index (1990–2012). *Source* KOF and SIPRI

**Fig. 11:**
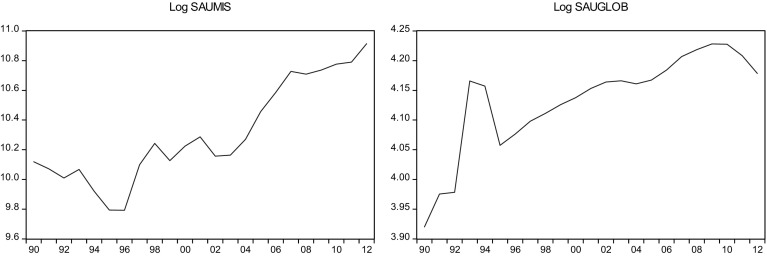
**a** Saudi Arabian military spending (1990–2012). **b** Saudi Arabian overall globalization index (1990–2012). *Source* KOF and SIPRI

**Fig. 12:**
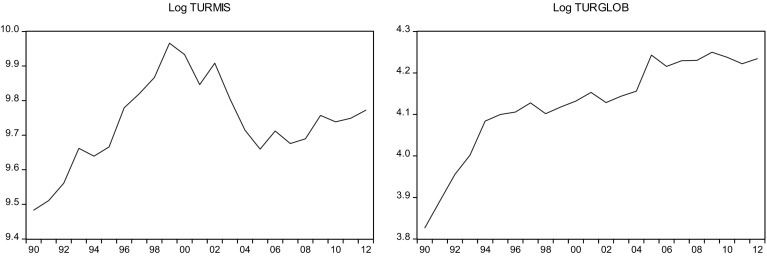
**a** Turkish military spending (1990–2012). **b** Turkish overall globalization index (1990–2012). *Source* KOF and SIPRI

**Fig. 13:**
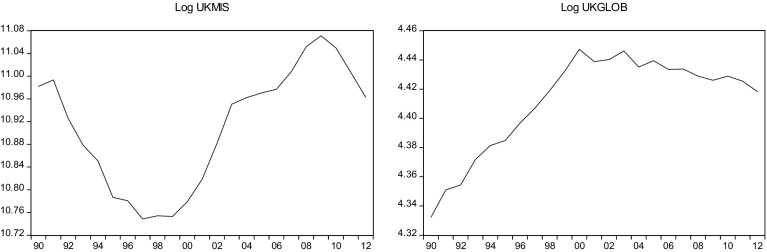
**a** British military spending (1990–2012). **b** British overall globalization index (1990–2012). *Source* KOF and SIPRI

**Fig. 14:**
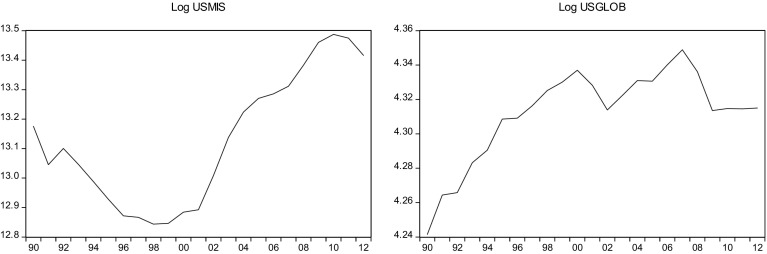
**a** US military spending (1990–2012). **b** US overall globalization index (1990–2012). *Source* KOF and SIPRI

### US

The $670 billion military expenditure (4.2% of GDP) put the US as a top spender on the list in 2012. Since 2001, US defense spending has risen from $397 billion to $670 billion. US military outlays fell from 4.6% of GDP in 2009 to 4.2% in 2012. Reduction in military expenditures was due to a greater focus on fiscal austerity and the diminishing the conflicts in Iraq and Afghanistan. In fact, military expenditure fell nearly 6% in 2012, followed by a 7.8% reduction in 2013.

While trade accounts for an increasing percentage of U.S. economic output (25%), U.S. trade as a percentage of GDP is lower than that of every other developed country in the world besides Japan. As the forces of globalization have reshaped the global economy, there has been increasing resistance to trade liberalization within the United States. They argue that free trade, which they view as unregulated, disenfranchises U.S. workers by outsourcing jobs overseas. Advocates assert that expanding free trade will create new U.S. jobs by opening up U.S. exports to a range of foreign markets, boosting competitiveness.

### China

Military spending in China was $19 and $161billion (2% of GDP) in 1990 and 2012, respectively. Military spending often reflects economic growth and this is especially true in China where military spending has increased in the past decades roughly in line with economic growth. Military expenditure grew 7.4% in 2013 alone, far more than any other country in the region, and among the larger annual growth worldwide. A combination of increased Chinese military spending and rising regional tensions have led to higher military expenditures among neighboring countries like Vietnam, Philippines, and Japan.

In China, the reforms mainly concerned the industrial sector and consisted of prices and wages liberalization, accompanied by the possibility of firms keeping the profits for self-financing. The increase in productivity and wages in this sector attracted labor force underemployed in the agricultural sector, contributing to the overall productivity growth. It was during 1990s that the “open door policy” started, thus supporting the beginning of integration of China into the world economy through both trade and FDI. Foreign firms were initially attracted by fiscal incentives. The gradual openness and extension of strong incentives to FDI was accompanied by persisting rigid conditions for admitting FDI.

### Russia

While total military spending in Russia remains a fraction of what it was in the late 1980s, it has been on the rise in recent years as a result of Russia´s involvement in regional conflicts, such as Ukraine crisis. The country´s military expenditure was roughly $81 billion in 2012 compared to just $64.5 billion in 2009. Russia now spends 4% of its GDP on its military. The rather high increase is likely due in part to Russia´s stated plans to invest more than $700 billion to modernize its weapons systems by 2020.

Foreign trade is very important for the Russian economy, which is open since 1990s. Openness combined with high levels of export and import concentration have made the country vulnerable to fluctuations abroad and to changes in the competitive environment, including: contractions in global oil and other commodity prices, exchange rate fluctuations of major currencies and contractions in major export markets. Policy makers attempt to pursue efforts towards increasing the geographical and product diversification of exports including through the support of the country’s quest for a modernization and “innovation based” model of development.

### Saudi Arabia

Saudi spent $24 and $55 (7.7% of GDP) on military expenditures in 190 and 2012, respectively. Located in an increasingly unstable region, Saudi Arabia increased its military budget by 14.3% in 2013. Saudi neighbors include Iraq, Syria, and Yemen, which are currently in turmoil. Saudi Arabia has also historically poor relations with another neighbor, Iran. The large increase in military outlays is likely a direct response to these threats. The country aims to replace its current 20-year old weapon stores, including a heavy investment in missile defense systems. Like many other countries with the biggest military budgets, Saudi Arabia benefits from one of the world´s largest oil reserves.

With regard to trade liberalization, the economy has had to be open to trade in order to act on its oil wealth. In the beginning of 1990, the country began negotiations to join the World Trade Organization (WTO), which ended in accession to the organization in 2005. As per the agreement with the WTO, Saudi Arabia agreed to reduce its tariffs. The agreement also provides market access for foreign services, including foreign insurance companies, banks, and telecommunications companies, all of which may now operate in the country subject to certain restrictions. Imports of merchandise goods have increased modestly since the agreement driven partly by the lower tariff rates, but service imports have risen substantially since 2005, probably as a result of this increased market access. Thus, Saudi Arabia’s economic policies and its outward-looking trade regime have enabled it to increase its trade openness and overall globalization.

### France

Like much of Western Europe, France´s military expenditure has decreased in recent years. France spent nearly $70 billion in 2009 versus $64 billion (2.3% of GDP) in 2012. This decrease, however, was relatively small given the country´s weak economic growth and implementation of the austerity measures after the global economic crisis. France passed the Military Programming Laws in 2013, which aims to keep the current level of military spending through 2019.

Most of France’s international trade is conducted with other European and, more specifically, European Union States, reflecting the progressive liberalization of the past thirty years undertaken in the context of European integration. The internationalization of the French economy over the past twenty years is revealed by the increase in the share of imports in domestic spending. EU and multilateral trade liberalization have played an important role in import penetration. However, France is characterized by a relatively low level of openness to international trade in goods and by a relatively low level of active internationalization.

### Brazil

Brazil spent about $19 billion and $38 billion on their military in 1990 and 2012, respectively, which amounted to 1.5% of the country’s GDP that year. Brazil is the only Latin American country with the ambition and resources to develop a diverse defense industrial base. Brazil’s 2008 National Strategy of Defense called for a robust domestic defense industry with the technological capacity to gradually rule out the need to purchase imported services and products. The construction of a Eurocopter plant and involvement of Brazilian firms in the construction of its Scorpene class submarines are as examples of recent efforts to implement this strategy. Furthermore, Brazil’s insistence on offsets and technology transfer as part of an upcoming fighter jet deal shows that further expansions to its defense industry are forthcoming. Although total defense spending in Brazil grew since 2006, it was a slightly decrease in military spending from 2011 to 2012. Whether this is an anomaly or a sign of longer-term reductions in Brazil defense spending aiming at reducing inflation and poverty, it is too soon to tell. However, high military spending can be controversial in the face of more pressing social needs. This tension has recently led to changes in budget priorities regarding military spending.

While Brazil has become one of the largest economies in the world, it remains among the most closed economies as measured by the share of exports and imports in GDP. This is due to a reliance on domestic value chain integration as opposed to participation in global production networks.

### United Arab Emirates (UAE)

The UAE armed forces have grown significantly over the years and are presently equipped with some of the most modern weapon systems, purchased from a variety of outside countries, mainly France, the US and the UK. A significant difference between the UAE and a number of other Gulf Cooperation Council (GCC) states is that its period of rapid defence spending growth came between 2007 and 2011. The UAE had the largest increases in military spending since 2005 which amounted to 135%. The total military spending was around $19 (4.8% of GDP) in 2012. UAE like Saudi Arabia is major oil producer, and their state revenues were boosted by high oil prices over the period. Military expenditure budgets of states in the region may be influenced by the fall in the price of oil in late 2014, but any impacts will likely be mitigated by the strong financial reserves built up by many countries following several years of high oil prices.

The UAE’s trade regime is open, with low tariffs and few non-tariff barriers to trade. The UAE’s openness was instrumental in order to promote its economic growth and facilitate the diversification of economic activity. The investment regime remains considerably more restrictive than the trade regime, as foreign participation in any domestic company or activity is limited to 49% of the capital; however, 100% foreign ownership is allowed in any of the UAE’s free zones. Improved market access for its products through multilateral trade liberalization and bilateral and regional trade agreements is a main trade policy objective.

### Turkey

Turkey spent $17 on their military in 2012 which amounted to 2.3% of the country’s GDP that year. The rather high military spending in Turkey stems from a perception that military power is a source of status. The military spending in Turkey was slightly declined due to the reduction in the intensity of the conflict with the Kurdistan Workers’ Party (PKK) during the past years but the recent conflict in Syria, Kurdistan, and the region might encourage an increase in military spending in Turkey. However, the high cost of maintaining a credible military establishment in an age of rapidly changing technology has incurred heavy expenditures by the Ministry of National Defence in relation to other demands on the government’s revenue. Consequently, the Turkish government has allocated funds to military in disproportion to widely acknowledged needs for social and economic development.

In 1990s and 2000s, the most important changes in the trade regime in Turkey were constituted by the Custom Union (CU) between the EU and Turkey and the consequent Free Trade Agreements (FTAs) signed with the European Free Trade Association countries, Israel, and the Central and Eastern European (CEE) countries. As a result of these changes in trade policy, the volumes of Turkish exports and imports increased substantially. At the same time, total FDI flows increased as well, both in absolute terms and as a share of GDP. However, the Turkish economy has become increasingly connected with the world market since 1990.

### UK

The military spending amounted to $58 (2.4% of GDP) in the UK in 2012. Like other countries in Western Europe, UK continued to cut military spending as austerity policies were maintained in most of the region. During 2013–2014 the military spending reduced by 2.5% but the government in the UK wants to raise it slightly due to the fight with IS. However, the UK government plans to continue the transformation of defence through the restructuring of the armed forces to create a simpler and more effective organization at a lower cost to the taxpayer.

The British economy’s openness to investment and trade has been a long-established fact, with many of the world’s largest firms having UK branch plants and manufacturing subsidiaries. The UK imposes few impediments to foreign ownership and throughout the past decade, the UK has remained Europe’s top recipient of FDI, including the destination of choice for U.S. investors. Although FDI has increased remarkably, in comparison with Europe, the UK is characterized by a relatively low level of openness to international trade in goods and by a relatively low level of active internationalization.

### Australia

The military spending was $26 (1.7% of GDP) in 2012. The budget delivers on the Government’s promise to grow, rather than cut, the defense budget. The Government remains firm on its commitment to increase defense spending to 2% of GDP within a decade. New projects will be announced in the Defense White Paper, which will be delivered later this year. The White Paper will outline the Government’s long-term defense strategy that will guide Australia’s defense capability over the coming decades. The Government continues to support the deployed defense force personnel including those in Iraq, Afghanistan, the Middle East and on maritime operations.

While Australia’s trade openness ratio is significantly below the average for developed countries, it is about the level that could be expected based on some major determinants of trade openness. The factors that best explain Australia’s relatively low openness are its remoteness from large economies and its large land mass. The first of these can be considered as a natural disadvantage, while the second can be regarded as an advantage since the natural diversity of its large land mass, Australia is able to produce many goods internally and does not need to trade for them externally. Australia’s relative proximity to India and China, and their strong trade links with them, suggests that Australia’s geographic location is likely to be less of a barrier to trade.

### Italy

From 2001 to 2012 the Italian total defense budget ranged from $17.2 billion to $35 billion (1.7% of GDP). In 2013 Italy planned to cut defense spending by 28%. Italy is facing economic constraints due to high fiscal debt, forcing the government to reduce its expenditure. This has generated constraints in the defense budget and reduced spending on the procurement of defense equipment. The economic crisis in Italy, and across Europe, led to reductions in the defense budgets of many countries which are some of the top importing countries of Italian defense products. The cuts will decrease the procurement expenditure of these countries and have a negative impact on the order book of the Italian defense industry. In addition, Italy’s membership of NATO, the UN, and the EU, and subsequent participation in peacekeeping operations, also require financial input.

Over the last decade, Italy has consolidated the internationalization process. However, there is still considerable scope for improvement. In comparison with Europe, Italy is characterized, in fact, by a relatively low level of openness to international trade in goods and by a relatively low level of active internationalization. Over the past 10 years, Italy’s limited growth has been the result of unsatisfactory productivity growth and lack of competition. For some years now there has also been an unexpected slowing of globalization, shown by the lower elasticity of international trade to output and by the deceleration of FDI. Furthermore, short-term factors, the phenomenon may also reflect a transition to a new phase of the international fragmentation of production, with an increase in the domestic value-added content of some value chains. The growth of international trade and investment is restrained by the persistence of significant tariff, quantitative and regulatory barriers. The multilateral trade negotiations are still far from a positive outcome.

### South Korea

The North Korean military is one of the biggest in the world, its defense budget would grow markedly over the next five years amid a growing perception of threats from North Korea. In 1990 and 2012, the military spending was $15 and $31 billion (2.6% of GDP), respectively. The annual rate of increase will be roughly 7% according to the Defense Ministry. Although the North Korean threat still justifies high military spending, other rationales have played a significant role in this development as well such as perception of weakening US security commitment, unspecified threats or insecurity in the region, the technological requirements of the Revolution in military affairs, and more importantly, arguing that growing the military and localizing production is good for the economy during the period of global economic crisis.

Regarding trade policy, the government switched its policy direction toward market openness, deregulation, and free trade during the early 1990s. It built on its stance of market openness and competition promotion continuously in the 2000s in order to expedite trade liberalization in pursuing free trade agreements with developing and developed economies around the world. This transformation was based on institutional changes to the trade policy-making setup in the beginning of the 21st century. At the same time, however, trade policy has been a very sensitive issue, and the government has struggled to ensure the greater inclusivity, transparency, and development of an effective safety net for disadvantaged sectors.

### Japan

The military spending was $47 and $60 billion (1% of GDP) in 1990 and 2012, respectively. Japan’s Defense Ministry has requested its biggest ever budget to boost its ability to protect outlying islands in response to China’s growing military reach in the region. The increase in the military expenditures reflects its growing anxiety about China’s expanding naval reach. The rise is also in line with Japan’s more assertive defense policy to check Chinese influence. The ministry is also seeking extra cash to build new military bases and expand existing ones on some of the islands, equipping them with state-of-the-art radar and missile batteries.

In terms of trade openness, Japan ranks relatively low to other countries. However, this trend is fairly typical of larger economies which tend to trade slightly less than smaller economies. In terms of FDI as a dimension of openness, Japan’s position is mixed. When evaluating both inward investment or liabilities and outward investment or assets, in terms of inward investment, Japan is relatively low, ranking the lowest among countries in the sample.

### Germany

Germany is to increase its defence spending, aiming to support NATO guidelines of spending 2% of GDP on national defence. In 2012, German military spending was $49 billion (1.4% of GDP). However, the defence budget is to rise by 6.2% over the next five years is a welcome recognition of the need for NATO countries not to drop their guard at a time of growing global instability. For obvious historic reasons, Germany’s defence spending has been relatively low over the past half-century compared with the size of its economy, the biggest in Europe. Its military is also constrained by the constitution from taking on overseas combat missions without parliamentary consent, though their military has been involved in a number of recent foreign operations. The German government plans to allocate additional funds to modernise the army and finance the growing engagement of German forces with NATO.

In 1995, the degree of openness of the German economy was lower than France’s, and also, albeit slightly, than Italy’s. Including intra-European trade, between 1991 and 2008, it increased from 52% to over 90% and became by far the highest of all the G7 countries, surpassing France and Italy by well over 50% and was nearly three times higher than America and Japan. The growing importance of Germany’s international activity was directly reflected in its GDP performance. While in the 1990s, foreign trade contribution to German economic growth was close to zero, starting in 1999, about 80% of it came from net exports. Since 2000, exports have grown by 7% in real terms per year. From that same year, Germany began to regain shares of world trade. The German foreign trade policy led to an increase in its trade openness and overall globalization index.

### India

The core message in the Indian Finance Minister’s statement is the push to become less dependent on foreign military know-how and imports and to revive the Indian defence industry. India is pursuing the “make in India” policy to achieve greater self-sufficiency in the area of defence equipment. The military spending was $49 billion (2.5% of GDP) in 2012. The high military spending in China, the border conflict with Pakistan, problems with Kashmiri insurgents are the main reasons for the increasing trend in military expenditures in India.

From mid-1991, the government of India introduced a series of reforms to liberalize and globalize the Indian economy. Reforms in the external sector were carried out to integrate the Indian economy with rest of the world. Reforms of trade and exchange rate policy were a critical element in the process of structural reform. Since the initiation of economic reforms, India’s outward orientation has increased remarkably. The major trade policy changes included simplification of procedures, removal of quantitative restrictions, and substantial reduction in the tariff rates. However, India’s approach to openness has been cautious, contingent on achieving certain pre-conditions to ensure an orderly process of liberalization and ensuring macroeconomic stability. Generally speaking, the policy regime in India with regard to liberalization of the external sector has witnessed perceptible change.

## Data and methodology

The data used in this study covers the period 1990–2012 for the top 15 military expenditure spenders (US, China, Russia, Saudi Arabia, France, UK, India, Germany, Japan, South Korea, Brazil, Italy, Australia, UAE, and Turkey). The chosen time period stems from the availability of data. The UAE was eliminated from the sample because of lack of data on military expenditures from 1990 to 1997. The variables used in this study include the real military expenditure (*MIS*), the overall globalization index (*GLOB*), and per capita real GDP (*GDPC*) as a control variable. The overall globalization index includes economic, social, and political globalization. The economic globalization (36%) consists of actual flows, trade and capital account restrictions. The social globalization (38%) involves data on personal contact, information flows, and cultural proximity. The political globalization (26%) consists of number of embassies in the country, membership in international organizations, participation in UN security-council missions, and international treaties. The variables are expressed in log forms. Data is obtained from the World Bank’s World Development Indicators, KOF Swiss Economic Institute’s overall globalization index, and Stockholm International Peace Research Institute (SIPRI).^3^

The estimation follows the bootstrap panel Granger causality proposed by Kónya (2006). This approach has two important advantages. First, it is not required to test the unit root and cointegration (i.e. the variables are used in their levels, without any stationarity conditions). Second, additional panel information can also be obtained given the contemporaneous correlations across countries (i.e. the equations denote a Seemingly Unrelated Regressions system- SUR system). Two steps should be followed before applying the bootstrap panel Granger causality: testing the panel for cross-sectional dependence and testing for cross-country heterogeneity. The first issue implies the transmission of shocks from one variable to others. In other words, all countries in the sample are influenced by globalization and have common economic characteristics. The second issue indicates that a significant economic connection in one country is not necessarily replicated by the others.

A set of three tests is constructed in order to check the cross-sectional dependence assumption: the Breusch and Pagan (1980) cross-sectional dependence (CD_BP_) test, the Pesaran (2004) cross-sectional dependence (CD_P_) test, and the Pesaran et al. (2008) bias-adjusted LM test (LM_adj_). Regarding the country-specific heterogeneity assumption, the slope homogeneity tests (${\bar{\Delta }}$ and $\mathop {{\bar{\Delta }}}\limits_{adj}$) of Pesaran and Yamagata (2008) are used. The Kónya’s (2006) approach considers both issues, based on SUR systems estimation and identification of Wald tests with country-specific bootstrap critical values. This procedure allows us to consider all variables in their levels and perform causality output for each country:

1 $$\begin{aligned} & GLOB_{1,t} = \alpha_{1,1} + \sum\limits_{i = 1}^{lm1} {\beta_{1,1,i} } GLOB_{1,t - i} + \sum\limits_{i = 1}^{\ln 1} {\delta_{1,1,i} } MIS_{1,t - i} + \sum\limits_{i = 1}^{lk1} {\gamma_{1,1,i} } GDPC_{1,t - i} + \varepsilon_{1,1,t} , \\ & GLOB_{2,t} = \alpha_{1,2} + \sum\limits_{i = 1}^{lm1} {\beta_{1,2,i} GLOB_{2,t - i} } + \sum\limits_{i = 1}^{\ln 1} {\delta_{1,2,i} } MIS_{2,t - i} + \sum\limits_{i = 1}^{lk1} {\gamma_{1,2,i} } GDPC_{2,t - i} + \varepsilon_{1,2,t} , \\ & \vdots \\ & GLOB_{N,t} = \alpha_{1,N} + \sum\limits_{i = 1}^{lm1} {\beta_{1,N,i} } GLOB_{N,t - i} + \sum\limits_{i = 1}^{\ln 1} {\delta_{1,N,i} MIS_{N,t - i} } + \sum\limits_{i = 1}^{lk1} {\gamma_{1,N,i} GDPC_{N.t - i} } + \varepsilon_{1,N,t} , \\ \end{aligned}$$

and

2 $$\begin{aligned} & MIS_{1,t} = \alpha_{2,1} + \sum\limits_{i = 1}^{lm2} {\beta_{2,1,i} } GLOB_{1,t - i} + \sum\limits_{i = 1}^{\ln 2} {\delta_{2,1,i} MIS_{1,t - i} } + \sum\limits_{i = 1}^{lk2} {\gamma_{2,1,i} GDPC_{1,t - i} } + \varepsilon_{2,1,t} , \\ & MIS_{2,t} = \alpha_{2,2} + \sum\limits_{i = 1}^{lm2} {\beta_{2,2,i} GLOB_{2,t - i} } + \sum\limits_{i = 1}^{\ln 2} {\delta_{2,2,i} MIS_{2,t - i} } + \sum\limits_{i = 1}^{lk2} {\gamma_{2,1,i} GDPC_{2,t - i} } + \varepsilon_{2,2,t} , \\ & \vdots \\ & MIS_{N,t} = \alpha_{2,N} + \sum\limits_{i = 1}^{lm2} {\beta_{2,N,i} GLOB_{N,t - i} } + \sum\limits_{i = 1}^{\ln 2} {\delta_{2,N,i} MIS_{N,t - i} } + \sum\limits_{i = 1}^{lk2} {\gamma_{2,N,i} GDPC_{N,t - i} } + \varepsilon_{2,N,t} . \\ \end{aligned}$$

In equation systems (1) and (2), *GLOB* is the overall globalization, *MIS* denotes the real military spending, *GDPC* is per capita real GDP as a control variable, *N* is the number of panel members, *t* is the time period (*t* = *1*,…, *T*), and *i* is the lag length selected in the system. The common coefficient is α, the slopes are *β*, *δ*, and *γ*, while ε is the error term. To test for Granger causality in this system, alternative causal relations for each country are likely to be found: (1) there is one-way Granger causality from X to Y if not all *δ*_*1,i*_ are zero, but all *β*_*2,i*_ are zero; (2) there is one-way Granger causality from Y to X if all *δ*_*1,i*_ are zero, but not all *β*_*2,i*_ are zero; (3) there is two-way Granger causality between X and Y if neither *δ*_*1,I*_ nor *β*_*2,i*_ are zero; and (4) there is no Granger causality between X and Y if all *δ*_*1,i*_ and *β*_*2,i*_ are zero. It is also allowed the maximal lags to differ across variables, but the same across equations. In this study, the system is estimated by each possible pair of *l*_*m1*_, *l*_*n1*_, *l*_*m2*_, *l*_*n2*_, *l*_*k1*_, and *l*_*k2*_, and it is assumed that 1–4 lags exist. Then the combinations that minimize the Schwarz Bayesian Criterion are chosen.

By inspecting the data, we find that most break dates correspond to major events such as the financial crisis of 1997–1998 and 2007–2008 and the economic downturn of 2001. Due to the existence of these structural breaks, we should incorporate these breaks into our testing model; otherwise, the results will be biased. Since Kónya (2006) cannot allow different break dates into the testing model, we follow the procedure adopted by Tsong and Lee (2011) and Bahmani-Oskooee et al. (2014) to adjust the data as follows:

3 $$\mathop {\hat{y}}\limits_{t} = y_{t} - \hat{\alpha } - \sum\limits_{l = 1}^{m + 1} {\mathop {\hat{\theta }}\limits_{l} } DU_{l,t} - \sum\limits_{i = 1}^{m + 1} {\mathop {\hat{\rho }}\limits_{i} } DT_{i,t} - \varepsilon_{t} ,$$

where, *ŷ*_t_ (either *GLOB* or *MIS*) is adjusted by the effect of possible structural breaks, *y*_*t*_ is *GLOB* or *MIS*, *DU*_t_ and *DT*_t_ are defined as the following:

4 $$DU_{k,t} = \left\{ {\begin{array}{*{20}l} 1 \hfill & {if\;TB_{k - 1} \prec t \prec TB_{k} } \hfill \\ 0 \hfill & {otherwise} \hfill \\ \end{array} } \right.,$$

5 $$DT_{k,t} = \left\{ {\begin{array}{*{20}l} {t - TB_{k - 1} } \hfill & {if\;TB_{k - 1} \prec t \prec TB_{k} } \hfill \\ 0 \hfill & {otherwise} \hfill \\ \end{array} } \right..$$

## Estimation results

Table 1 reports the results of cross-sectional dependence tests (CD_BP_, CD_p_, and LM_adj_) and slope homogeneity tests (${\bar{\Delta }}$ and $\mathop {{\bar{\Delta }}}\limits_{adj}$). The first set of tests, for cross-sectional dependence, clearly reveals that the null hypothesis of no cross-sectional dependence is rejected for all significance levels. More precisely, this implies that there is a cross-sectional dependence in the case of our sample countries. Any shock in one country is transmitted to others, the SUR system estimator being more appropriate than country-by-country pooled OLS estimator. The second part of the table shows that the null hypothesis of slope homogeneity is rejected for both tests and for all significance levels. In this case, the economic relationship in one country is not replicated by the others. As there are both cross-sectional dependence and slope heterogeneity, the bootstrap panel Granger causality approach can be applied.

**Table 1:** Cross-sectional dependence and slope homogeneity tests

Method	Test statistics	*p* value
Cross-sectional dependence test		
CD_BP_	213.724***	0.0000
CD_P_	15.155***	0.0000
LM_adj_	28.316***	0.0000
Slope homogeneity test		
${\bar{\Delta }}$ test	25.230***	0.0000
$\mathop {{\bar{\Delta }}}\limits_{adj}$ test	21.597***	0.0000

CD_BP_ test, CD_P_ test, and LM_adj_ test show the cross-sectional dependence tests of Breusch and Pagan (1980), Pesaran (2004), and Pesaran et al. (2008), respectively. ${\bar{\Delta }}$ test and $\mathop {{\bar{\Delta }}}\limits_{adj}$ test show the slope homogeneity tests proposed by Pesaran and Yamagata (2008)

*** Significance for 0.01 levels

The results of the bootstrap panel Granger causality test are shown in Table 2. The findings show that *GLOB* and *MIS* are causally related in most of the countries under review. There is a bi-directional causality in UK, US, Saudi Arabia, and Russia. The causality is unidirectional running from *GLOB* to *MIS* in Australia, Brazil, India, and China, and running from *MIS* to *GLOB* in Turkey. The degree of significance level varies from country to country. There is no any causal relationship between military spending and globalization in France, Italy, South Korea, Germany, and Japan. Overall, this evidence shows a relatively robust association between changes in globalization and changes in military expenditure. In other words, countries experiencing greater globalization have relatively large increases in militarization over the past 20 years.

**Table 2:** The bootstrap panel Granger causality results

Country	H0: *GLOB* does not Granger cause *MIS*				H0: *MIS* does not Granger cause *GLOB*			
	Wald test	Bootstrap critical value			Wald test	Bootstrap critical value		
		1%	5%	10%		1%	5%	10%
Germany	3.679	15.523	8.675	5.436	4.722	13.752	7.824	4.935
France	0.985	18.637	9.574	6.566	3.546	10.738	6.275	4.012
Italy	12.143	35.728	18.672	13.233	7.541	32.484	17.317	12.924
China	14.982**	19.328	9.807	7.125	4.781	19.097	10.267	7.453
India	33.526**	44.162	25.252	19.419	15.306	43.357	23.456	19.182
Brazil	11.425**	20.713	10.756	8.386	5.597	18.649	9.847	7.252
Russia	20.534**	21.274	12.961	9.705	16.203*	35.438	18.758	13.353
Turkey	3.253	15.677	8.752	6.381	8.385*	17.231	9.692	7.075
Australia	21.737*	48.259	25.654	20.286	13.838	34.628	18.219	14.762
UK	29.340**	46.369	25.452	19.013	25.230**	30.372	16.567	12.313
US	24.675**	27.654	14.055	10.729	10.985*	24.362	13.179	10.591
South Korea	8.338	25.632	13.728	9.815	9.312	27.221	14.576	11.212
Saudi Arabia	13.046*	26.975	14.281	11.176	65.185***	54.271	29.392	22.599
Japan	17.459	52.493	27.311	21.239	12.294	39.672	19.237	14.538

Bootstrap critical values are obtained from 10,000 replications

*, **, and *** significance at the 0.10, 0.05, and 0.01 levels, respectively

However, it has been shown that globalization may not lead to more peaceful relations or demilitarization. As we discussed in Sect. 2, bilateral trade increases the opportunity cost of bilateral war and may hinder bilateral war. Globalization (equivalent to multilateral economic openness) reduces this opportunity cost with any given country and devitalize the incentive to make concessions during negotiations, and, therefore, increases the probability of war between any given pair of country. Thus, an increase in trade or openness between two countries may restore peace between those but may increase the probability of conflict with third countries.

## Conclusion

While previous studies mostly focused on the causal nexus between military expenditure and economic growth, those studies have not considered the role of globalization. This study uses data from the top 15 military expenditure spenders over the period 1990–2012 to examine the relationship between militarism and globalization. The bootstrap panel Granger causality that accounts for both cross-sectional dependence and heterogeneity across countries is utilized to detect the direction of causality. The results show that military expenditures and globalization are causally related in most of the countries under review.

Despite the increasing role of globalization, the results show that military expenditures are growing and pointing to a strengthening in nationalist sentiments and militarism. This paper suggests that changes in domestic political and economic conditions might hinder the process of globalization. The results are consistent with those of Acemoglu and Yared (2010) who conclude that high military spending endangers globalization. This study also supports the results of Martin et al. (2008) who find that an increase in multilateral trade raises the chance of conflict between states. The policy implication of the findings is that greater military spending by a country increases the likelihood of military conflict in the future, the anticipation of which discourages globalization.

## Footnotes

1 Mansfield and Pollins (2006) provide an excellent survey on the theoretical relationship between militarism and economic interdependence. Morrow (2000) provides a game theoretical approach to globalization and militarism.

2 In this section data on military spending is in terms of constant prices and comes from SIPRI. Data on the overall globalization index is obtained from KOF Swiss Economic Institute. Any other statistics is obtained from the World Bank’s World Development Indicators.

3 Military expenditure in 1990 and 1991 is missing for Russia, so we use the military spending from 1992 for both observations.
